# Supplementary material for: Under-five mortality and associated factors in southeastern Ethiopia
Source: PLoS One. 2021 Sep 7;16(9):e0257045. doi: 10.1371/journal.pone.0257045 (PMC8423275; doi:10.1371/journal.pone.0257045)
Supplement: Attachment — Submitted filename: rew.pdf [file pone.0257045.s002.pdf]

It’s a good paper with surprising numbers in respect with European situation.

The authors should better explicit the Ethiopian health system, how the children arrive in the hospital and, for example, how long is the mean waiting time before to be visited from a medical doctor.

Particularly should be clarified the prevalence of HIV in Etiopia in the general population and in the children population or prevalence of TBC pneumonia.

The authors should specify when the therapy for pneumonia is established during the first 48 hours and specify if it was oxygen therapy or IV antibiotics.
