# Supplementary material for: Under-five mortality and associated factors in southeastern Ethiopia
Source: PLoS One. 2021 Sep 7;16(9):e0257045. doi: 10.1371/journal.pone.0257045 (PMC8423275; doi:10.1371/journal.pone.0257045)
Supplement: Attachment — Submitted filename: Response to reviewers.docx [file pone.0257045.s003.docx]

**Response to editor and reviewers’ comments:**

**June1, 2021**

Dear Dr Tai-Heng Chen, Academic editor of PLOSONE, we thank you and the reviewers for a thorough reading and constructive comments on our manuscript entitled ‘’*Under-five mortality and associated factors in southeastern Ethiopia*’’. These comments are all valuable and very helpful for revising and improving our paper. We have tried our best to revise our manuscript according to reviewers’ comments. We are pleased to submit the improved research article. Revised portions are marked in red in the Manuscript with Track changes. The responses to the reviewers’ comments are listed as follows:

**Editor:**

The three reviewers, in the ‘’reviewers’ response to questions ‘’ agreed it is a good manuscript, focused on addressing of an important problem. But our manuscript has weaknesses that the three reviewers’ thinks should be addressed.

**Response:** Thank you very much for your hard work. We have tried our best to revise our manuscript according to reviewers’ comments and marked in red in the Manuscript with Track changes.

**Response to Reviewer #1:**

**Point 1:** The number of under-5 deaths globally needs to be either updated or cited correctly.

**Response 1:** Thank you very much for your suggestion. As per your recommendation, we have updated with the recent Global Burden of Disease study from 2019.

Reference: GBD 2019 Demographics Collaborators. Global age-sex-specific fertility, mortality, healthy life expectancy (HALE), and population estimates in 204 countries and territories, 1950-2019: a comprehensive demographic analysis for the Global Burden of Disease Study 2019. Lancet, 2020 Oct 17;396 (10258):1160-1203. doi: 10.1016/S0140-6736(20)30977-6. PMID: 33069325; PMCID: PMC7566045.

**Point 2:** Define what is meant by “early child mortality” used in paragraph 3.

**Response 2:** We apologize for not stating it clearly. We have corrected and substituted it with early child death (death within the first ≤24 hours of arrival in the hospital).

**Point 3:** Paragraph 4: It’s not clear what the authors mean exactly by “the limited availability of data” as the prior 3 paragraphs provide estimates on under-5 mortality and even data on childhood mortality trends in Ethiopia.

**Response 3:** We mean limited availability of data in the region. By taking the comment into consideration, we have modified it as; Despite the achievement, the limited availability of data in southeastern Ethiopia poses a considerable challenge on generating reliable estimates of under-five mortality in that particular area.

**Point 4:** I suggest the authors remove the mention of the period in which the study was conducted (i.e., August-November 2019) as it creates confusion. The study period appears to be Sept. 2014-July 2019 in paragraph 2.

**Response 4:** We have accepted the suggestion and modified it accordingly. A register based cross sectional study was conducted from September 2014 to July 2019 in Asella teaching and referral hospital……

**Point 5:** What do the authors mean by “complete information” in paragraph 2 of the Methods?

**Response 5:** We have indicated the explanation for complete information in paragraph 2 of the methods. It means data filled with all information on the registration book. If the data is not fully filled, we have excluded them from our study.

**Point 6:** The authors mention that patients without complete data were excluded. I suggest they mention what kind of bias may have been introduced in this in the Limitations.

**Response 6:** Thank you for the suggestion. We have indicated the exclusion of 14 under-five children with incomplete data in the first paragraph of our result section.

**Point 7:** Did the authors electronically transcribe what was handwritten in the registry at this hospital? This needs to be clarified.

**Response 7:** Yes. As per your suggestion, we have tried to clarify it in data processing and analysis section. We have prepared structured data sheet to extract data of the eligible cases from the registration book of pediatrics ward and the collected data on the sheet from the hand written hospital registry was entered into Epidata version 7. Finally, the analysis was carried out using SPSS version 21.

**Point 8:** I suggest the authors provide some rationale for the selection of their independent variables. Was there previous literature upon which the authors decided to select these candidate variables? Was there some clinical rationale?

**Response 8:** Thank you for the suggestion. We came to understand the confusion it creates and indicated the way we selected our independent variables. All were taken from registration book

**Point 9:** What exactly is a “National classification of disease diagnosed”?

**Response 9:** We have tried to define what is meant by National classification of disease diagnosed in our operation definition part. It means primarily diagnosed diseases at the time of admission in the hospital. It contains all diagnosed cases like SAM, measles, meningitis, acute gastro-enteritis with dehydration, surgical conditions and others.

**Point 10:** It appears the authors do not include all independent variables in their list. Specifically, they say malnutrition was associated with mortality in the abstract, but make no mention of that in the independent variables.

**Response 10:** Malnutrition and other primarily diagnosed diseases at the time of admission in the hospital were all under national classification of diseases. We have taken your comment and mentioned all the primary diagnosis in the independent variable section.

**Point 11:** I’m not sure of the rationale for including the difference between the various definitions of mortality in the Operational definitions section. I suggest the authors weave these into their variable definitions instead.

**Response 11:** Thank you for your suggestion. We have excluded the various definitions of mortality from the operational definition section and included definitions of variables used in our study.

**Point 12:** It is highly unusual to include subheadings like “Competing interests” and “Acknowledgements” in the Methods. These are usually placed at the end of the manuscript.

**Response 12:** We apologize for our negligence. We have placed it at the end of the manuscript.

**Point 13:** If the authors only included patients who had complete data, how many were excluded from their analysis? Can they say how these differed from the study population? What bias was introduced in excluding those patients? Meaning, how may they have been different than the patients included?

**Response 13:** We have indicated the exclusion of 14 under-five children with incomplete data. A total of 4915 children were admitted in the hospital among which the data of 14 were not registered completely. Since the number of excluded individuals were too small, we believe it have no influence over the overall result.

**Point 14:** How were “urban” and “rural” defined in Table 1? This should be defined in the Methods.

**Response 14:** Thank you for your suggestion. We have defined it in operational definition part. Urban residents are defined as individuals living in cities (which have a population of at least 50,000 inhabitants in contiguous dense grid cells with >1,500 inhabitants per km^2^) and towns which have a population of at least 5,000 inhabitants in contiguous dense grid cells with >300 inhabitants per km^2^), and Rural residents are individuals residing mostly of low-density grid cells.

**Point 15:** Table 1: What does “Referred to higher” mean? It seems to be an incomplete phrase. Also, how were the “conditions at discharge” defined? Should be included either as a footnote to the Table or in the Methods.

**Response 15:**  We have indicated the discharge condition on the methods part. We have taken discharge conditions on the registration book which were classified as Alive (Improved, Same, Left Against Medical (Self-discharge), Referred to higher (Referred for diagnostic or therapeutic services that are not available at the hospital OR referred because the hospital beds are all occupied) and Absconded (Escaped)) and Died.

**Point 16:** For Table 2, did the authors account for multiple diagnoses for each patient? Are these primary diagnoses?

**Response 16:** No, they all are primary diagnosis at the time of admission. We have indicated in the description.

**Point 17:** Table 2: What is HAAD?

**Response 17:** We apologize for putting it as it is. We have indicated what HAAD (Hyper-Active Airway Disorder) is.

**Point 18:** I think Table 3 could be summarized in the text. I’m not sure what the value is in seeing the differences in the patient’s sex as this does not seem to be a focus of the analysis at any point.

**Response 18:** Thank you for the suggestion. We have excluded the differences in the patient’s sex from the table but, since prevalence of under-five, post-neonatal and child mortality is one of the main focus of the study, we believe it will be better if it appears with the description.

**Point 19:** Figure 1 needs some sort of scale or dots to indicate what the difference size of the lines means. It might be better displayed as a bar graph with the proportion of mortality at each discharge interval shown.

**Response 19:** We have appreciated your observation and replaced it with bar graph.

**Point 20:** Table 4: The variable shouldn’t be called “address”. Urbanicity would be more fitting.

**Response 20:** Thank you for the suggestion. We have modified it as place of residence throughout the document.

**Point 21:** How was shock defined in this study? This needs to be clearly spelled out, particularly given the potential for subjectivity with this diagnosis. Also, what type of shock? Hypovolemic is much different than cardiogenic, for example.

**Response 21:** It is hypovolemic shock. We have tried to correct it as per your suggestion throughout the document.

**Point 22:** Paragraph 1 should not restate specifics of the Results section. Instead, I suggest the authors state in broad strokes their main findings here.

**Response 22:** Thank you, we have considered your nice view. We have restated it as; Given that infant and child mortality rates are the basic indicators of a country’s socioeconomic situation and quality of life, this study was conducted to assess the prevalence and associated factors for under-five, child and post-neonatal mortality in Asella teaching and referral hospital, southeastern Ethiopia. The study revealed the prevalence of 8.7% (87/1000) (95% CI, 7.91%-9.50%) under-five mortality. Post-neonatal and child mortality rates were 9.1% (91/1000) and 8.18% (81.8/1000), respectively. Place of residence, HIV status, severe acute malnutrition, hypovolemic shock, type I diabetes with DKA and length of stay in the hospital for ≤2 days and 3-4 days were among the identified predictors.

**Point 23:** If the authors state that mortality could be higher as this study was conducted at a referral hospital, do they have any data on referral status to justify this statement? Could that have been an important covariate to include?

**Response 23:** We don’t have data on referral status. That is why we justified it with ‘’might be attributed……….., taking the health system in the country into consideration’’.

**Point 24:** The discussion about HIV is very superficial. Can the authors say anything about whether or not the patients who had HIV were on antiretroviral therapy? Was that a covariate that could have been an important factor in HIV-related mortality?

**Response 24:** Thank you very much for your comment. We have tried to modify it as; Even though starting ART instantly to all children living with HIV regardless of their WHO clinical stages and CD4 counts/percentage is recommended and being implemented, our study depicted HIV as a significant predictor for under-five mortality. This finding was consistent with a result from cohort study in Kenya and South Africa in which HIV infection was associated with increased mortality. The evidence suggests that HIV-infection may increase susceptibility to different diseases and greatly impact the health of HIV-infected children, which subsequently can increase the occurrence of under-five mortality. Among the participants, our study found HIV prevalence of 0.8%, nearly close to the nationwide HIV prevalence (0.9%). The data obtained from UNAIDS in 2019 also showed that approximately 44,000 children less than 14 years lived with HIV.

We have used additional references:

- Federal Ministry of Health of Ethiopia, National consolidated guidelines for comprehensive HIV prevention, care and treatment. August 2018.
- UNAIDS DATA 2020, <https://www.unaids.org/sites/default/files/media_asset/2020_aids-data-book_en.pdf>. Accessed May, 2021

**Point 25:** In general, I think the authors could improve the Discussion by including more of the implications of their findings as opposed to simply comparing and contrasting to existing literature on the topic. For example, if early mortality is a problem, what specific interventions could be employed in the future to reduce such deaths? If HIV is a problem, what could be done to reduce these deaths?

**Response 25:** Thank you for your suggestion. We have tried to modify the discussion and recommendation thoroughly as per your suggestion.

**Point 26:** In the limitations paragraph, what do the authors mean exactly by “under-five children admitted outside the pediatric ward”. Where were those children admitted?

**Response 26:** Children’s admitted in pediatric emergency unit of the hospital. In our setup, children’s who visited the hospital in time other than working hours or came with emergency conditions will be seen at pediatric emergency units. Those improved will be discharged (these are the one excluded from the study) to their home and those who fulfills the admission condition will be admitted the pediatric ward of the hospital.

**Response to Reviewer #2:**

In this manuscript, “Under-Five Mortality and Associated Factors in Southeastern Ethiopia,” the authors were aim to estimate the overall prevalence and associated risk factors of mortality among under-five children and post-neonates in southeastern Ethiopia. However, I feel that some adjustments and clarifications are strongly needed.

**Point 1:** First of all, the manuscript is not well structured. The tables and figures did not present the reasonable results according to the study aim. The authors should think how to present the percentage in your tables. Then, the meaning of Figure1 was not clear.

**Response 1:** Thank you for your comment. Considering it, we have tried to well structure our manuscript. We have also tried to modify our tables in order to present our results according to the study aim. In addition, we have omitted Figure 1 and replaced it with bar graph.

**Point 2:** In addition, the section of method was too weak. The authors should follow the STROBE statement to describe the study design, setting, how to select the participants, all interested variables, potential bias, the sample sizes and power, and what statistical methods used.

**Response 2:** Thank you for your suggestion. Taking your suggestion into consideration, we have tried to improve our method section.

**Point 3:** Finally, the authors did not indicate their study limitation.

**Response 3:** We have indicated the limitation of our study at the last paragraph of the discussion. Here is what we have written: We acknowledge that our study has a number of caveats. To mention some, the finding should be considered cautiously while interpreting since the data were retrospectively extracted from hand written medical records. In addition, under-five children admitted outside the pediatric ward of the hospital were not included in the study and the cause for under-five mortality made by the health care providers may not accurately reflect the entire cause of death provided that autopsy is not performed.

**Response to Reviewer #3:**

It’s a good paper with surprising numbers in respect with European situation.

**Point 1:** The authors should better explicit the Ethiopian health system, how the children arrive in the hospital and, for example, how long is the mean waiting time before to be visited from a medical doctor.

**Response 1:** Thank you for your comment. As we have tried to indicate in the manuscript, we have collected the data from hand written registration book in the pediatrics of the hospital. Both the hospital registry and patient card didn’t have any data with regard to the point raised. So, it is difficult to clearly indicate the mean waiting time before to be visited from medical doctor.

**Point 2:** Particularly should be clarified the prevalence of HIV in Ethiopia in the general population and in the children population or prevalence of TBC pneumonia.

**Response 2:** Thanks for the suggestion. We have tried to discuss more on HIV prevalence in adult and children population using different references. Here is our modification on that particular area:

Even though starting ART instantly to all children living with HIV regardless of their WHO clinical stages and CD4 counts/percentage is recommended and being implemented, our study depicted HIV as a significant predictor for under-five mortality. This finding was consistent with a result from cohort study in Kenya and South Africa in which HIV infection was associated with increased mortality. The evidence suggests that HIV-infection may increase susceptibility to different diseases and greatly impact the health of HIV-infected children, which subsequently can increase the occurrence of under-five mortality. Among the participants, our study found HIV prevalence of 0.8%, nearly close to the nationwide HIV prevalence (0.9%). The data obtained from UNAIDS in 2019 also showed that approximately 44,000 children less than 14 years lived with HIV.

**Point 3:** The authors should specify when the therapy for pneumonia is established during the first 48 hours and specify if it was oxygen therapy or IV antibiotics.

**Response 3:** Thank you for your comment. As stated above, we have used secondary data from the hospital. Unfortunately, the time as to when the therapy for pneumonia is established is not documented on the hospital registry and What is available on the registration book is the primary diagnosis and the outcome of the child. The way they managed each primary diagnosis is not included on the document.

On behalf of my co-authors, I thank you for your consideration of this resubmission. We appreciate your time and look forward to your response.

Best,
