# Supplementary material for: Under-five mortality and associated factors in southeastern Ethiopia
Source: PLoS One. 2021 Sep 7;16(9):e0257045. doi: 10.1371/journal.pone.0257045 (PMC8423275; doi:10.1371/journal.pone.0257045)
Supplement: Attachment — Submitted filename: Response to reviewers.docx [file pone.0257045.s004.docx]

**Response to editor and reviewers’ comments:**

**July 21, 2021**

Dear Dr Tai-Heng Chen, Academic editor of PLOSONE, we thank you and the reviewers for the second round constructive comments on our manuscript entitled ‘’*Under-five mortality and associated factors in southeastern Ethiopia*’’. The comments are all valuable and very helpful for revising and improving our paper. We have tried our best to revise our manuscript according to the comments. We are pleased to submit the improved research article. Revised portions are marked in red in the Manuscript with Track changes. The responses to the reviewers’ comments are listed as follows:

**Editor:**

The reviewers have appreciated the authors for being very responsive to their comments and appreciated the significant improvement on the manuscript. But the reviewers have some minor comments that should be addressed.

**Response:** Thank you very much for your hard work. We have tried our best to revise our manuscript according to reviewers’ comments and marked in red in the Manuscript with Track changes.

We have uploaded our figure file to the Preflight Analysis and Conversion Engine (PACE) digital diagnostic tool to fit with the requirement of the journal and ensure that all our reference lists are complete and correct.

**Response to Reviewer #1:**

**Point 1:** I think the authors could provide more of a rationale beyond "the variables were in the registry" as to why they were selected as predictors of mortality. There is likely other literature the authors can refer to in the methods to indicate why things like SAM and HIV were thought to contribute to mortality. The same applies to length of stay.

**Response 1:** Thank you very much for your suggestion. We have tried to provide rationale for each stated predictors of under-five mortality. For example; HIV was one of the factors identified as a predictor for under-five mortality in our study. We have tried to justify as to why it is a predictor as ‘’the evidence suggests that HIV-infection may increase susceptibility to different diseases and greatly impact the health of HIV-infected children, which subsequently can increase the occurrence of under-five mortality’’.

For Severe Acute Malnutrition, we have indicated as ‘’the high mortality occurring with severe acute malnutrition or shock could be explained by the poor reaction of the body that has suffered reductive adaptation to the medical intervention at the time of admission in the hospital’’, and for the length of stay in the hospital ‘’this doesn’t mean length of admission in the hospital is a predictor of mortality but rather, delays in care seeking by family members and late referral of the sick children might contribute for higher death in early days of admission. In addition, the priority and quality of emergency care given to the cases in the hospital might also be a reason’’.

**Point 2:** I think the authors should mention in the Discussion or the Limitations that they did not account for any gaps in the quality of clinical care that may have contributed to such high rates of mortality.

**Response 2:** We have accepted the suggestion and mentioned it in the limitation section as ‘’Moreover, our study did not account for any gaps in the quality of clinical care which may have contributed to high rates of mortality’’.

**Response to Reviewer #3:**

**Point 1:** "from 1^st^ August to 30^th^ November, 2019 in ..." in abstract should be changed in: from 1^st^ September 2014 to July 2019

**Response 1:** Thank you for your suggestion. We have corrected it.

On behalf of my co-authors, I thank you for your consideration of this resubmission. We appreciate your time and look forward to your response.

Best,
